# Supplementary material for: Clinical, Psychosocial, and Structural Factors Associated with the Detection of HIV Drug Resistance in Children Living with HIV in Kisumu, Kenya: Secondary Analysis of Data from the Opt4Kids Study
Source: Viruses. 2025 Sep 16;17(9):1246. doi: 10.3390/v17091246 (PMC12474375; doi:10.3390/v17091246)
Supplement: Supplementary file 1 [file viruses-17-01246-s001.zip › viruses-3808909-supplementary.pdf]

Table S1. Factors associated with a drug resistance penalty score (DR-PS) of < 15 versus a DR-PS ≥ 15 and DR-PS < 60 versus DR-PS ≥ 60 to any antiretroviral agent assessed by Stanford HIVdb that was detected in children enrolled in Opt4Kids studies (*n*=704).

	**Penalty score ≥15 (*n*=97)**		**Penalty score ≥60 (*n*=91)**	
	Unadjusted RR[1]	Adjusted RR[1]	Unadjusted RR[1]	Adjusted RR[1]
Child Clinical Characteristics				
Age category (years)				
1–5	2.46 (1.58, 3.83)	1.21 (0.46, 3.18)	2.81 (1.78, 4.44)	1.84 (1.07, 3.14)
6–10	0.89 (0.58, 1.37)	0.74 (0.48, 1.13)	0.98 (0.63, 1.55)	0.77 (0.51, 1.16)
11–15 (Ref)	1.0	1.0	1.0	1.0
Sex				
Male (Ref)	1.0		1.0	
Female	1.07 (0.74, 1.55)		1.02 (0.7, 1.5)	
History of VF within two years prior to study				
Yes	6.55 (4.41, 9.72)	4.30 (2.76, 6.71)	7.26 (4.77, 11.05)	4.18 (2.77, 6.31)
No (Ref)	1.0	1.0	1.0	1.0
Base ART regimen at index DRM result				
NNRTI-containing (Ref)	1.0	1.0	1.0	1.0
PI-containing	2.66 (1.64, 4.32)	2.01 (1.16, 3.51)	3.02 (1.81, 5.04)	1.83 (1.08, 3.11)
INSTI-containing	8.70 (5.40, 14.0)	6.27 (3.40, 11.54)	9.38 (5.61, 15.68)	6.05 (3.43, 10.68)
Only NRTI-containing[2]	-		0.12 (0.02, 0.90)	-
Co-administered NRTIs with base ART regimen at index DRM result[3]				
ABC+3TC (Ref)	1.0		1.0	
AZT+3TC	1.24 (0.78, 2.0)		1.36 (0.85, 2.20)	
TDF+3TC	1.87 (1.18, 2.95)		1.94 (1.21, 3.13)	
Caregiver Clinical Characteristics				
Age of caregiver				
<24 years	2.16 (1.12, 4.16)	1.21 (0.65, 2.25)	2.52 (1.29, 4.92)	1.10 (0.64, 1.87)
24-35 years	1.57 (1.05, 2.33)	1.29 (0.82, 2.03)	1.79 (1.17, 2.73)	1.28 (0.86, 1.91)
>35 years (Ref)	1.0	1.0	1.0	1.0
Caregiver viral suppression				
Yes (Ref)	1.0	1.0	1.0	1.0
No	2.05 (1.13, 3.69)	1.04 (0.52, 2.06)	1.95 (1.04, 3.66)	1.37 (0.74, 2.54)
Not applicable	1.18 (0.74, 1.90)	1.23 (0.77, 1.97)	1.19 (0.73, 1.94)	1.21 (0.80, 1.83)
Unknown	1.05 (0.60, 1.84)	1.39 (0.74, 2.61)	1.02 (0.57, 1.85)	1.42 (0.83, 2.45)
Child Psychosocial Characteristics				

School level				
Nursery (Ref)	1.0			
Primary	0.87 (0.53, 1.43)		0.80 (0.48, 1.32)	
Secondary[2]	-		-	
School type				
Day school	0.85 (0.14, 5.31)	0.75 (0.09, 6.59)	0.80 (0.13, 4.97)	
Boarding[2]	0.02 (0.0, 0.13)	-	-	
Mix of day and boarding (Ref)	1.0	1.0	1.0	
Adherence				
Good (Ref)	1.0	1.0	1.0	1.0
Poor	2.06 (1.42, 3.0)	2.09 (1.38, 3.17)	2.17 (1.48, 3.2)	1.91 (1.32, 2.76)
Adolescence awareness of status				
No	1.08 (0.74, 1.57)		1.11 (0.75, 1.64)	
Yes (Ref)	1.0		1.0	
Caregiver Psychosocial Characteristics				
Caregiver marital status				
Married	1.0		1.2 (0.8, 1.81)	
Unmarried (Ref)	0.87 (0.53, 1.43)		1.0	
Unknown[2]	-		-	
Caregiver educational attainment				
No Education	0.48 (0.12, 1.88)		0.52 (0.13, 2.03)	
Primary	0.96 (0.66, 1.4)		0.98 (0.66, 1.45)	
Secondary & above (Ref)	1.0		1.0	
Caregiver depression				
Yes	1.37 (0.94, 1.98)	0.96 (0.61, 1.50)	1.34 (0.91, 1.97)	1.02 (0.70, 1.49)
No (Ref)	1.0	1.0	1.0	1.0
Caregiver stigma				
Yes	1.03 (0.61, 1.73)		0.86 (0.48, 1.53)	
No (ref)	1.0		1.0	
Caregiver HIV literacy				
Yes (Ref)	1.07 (0.74, 1.55)		0.99 (0.67, 1.45)	
No	1.0		1.0	
Caregiver medicine administration confidence				
Yes (Ref)	1.0	1.0	1.0	1.0
No	1.96 (1.11, 3.46)	1.93 (1.01, 3.67)	2.11 (1.19, 3.73)	1.89 (1.11, 3.22)
Caregiver IPV				
Yes	1.15 (0.76, 1.75)		1.12 (0.72, 1.74)	
No (Ref)	1.0		1.0	
Household Psychosocial Characteristics				

Household food insecurity				
No (Ref)	1.0		1.0	
Yes	1.11 (0.30, 4.10)		2.1 (0.31, 14.12)	
Other children in the household				
Yes	1.45 (0.70, 3.00)		1.35 (0.65, 2.81)	
No (Ref)	1.0		1.0	
Other CLHIV in the household				
Yes (Ref)	1.0		1.0	
No	1.15 (0.67, 1.99)		1.30 (0.71, 2.35)	
Structural Characteristics				
Time to facility				
< 30 minutes (Ref)	1.0	1.0	1.0	1.0
30 minutes to 1 hour	0.54 (0.36, 0.83)	0.75 (0.47, 1.19)	0.55 (0.36, 0.85)	0.72 (0.47, 1.12)
> 1 hour	0.67 (0.42, 1.09)	0.68 (0.39, 1.21)	0.60 (0.36, 1.01)	0.71 (0.44, 1.14)
Clinic location				
Rural	0.77 (0.44, 1.36)		0.75 (0.41, 1.35)	
Semi-urban	1.26 (0.83, 1.92)		1.23 (0.8, 1.91)	
Urban (Ref)	1.0		1.0	
Clinic volume				
Light (Ref)	1.0		1.0	1.0
Medium	0.59 (0.35, 0.98)	0.50 (0.28, 0.87)	0.54 (0.32, 0.92)	0.55 (0.33, 0.92)
Heavy	0.55 (0.30, 0.98)	0.58 (0.29, 1.16)	0.50 (0.28, 0.91)	0.76 (0.41, 1.39)

Abbreviations: ABC—Abacavir; ART—antiretroviral therapy; AZT—Zidovudine; CLHIV—children living with human immunodeficiency virus; NRTI—nucleoside reverse transcriptase inhibitor; NNRTI—non-nucleoside reverse transcriptase inhibitor; INSTI—integrase strand transfer inhibitor; IPV—intimate partner violence; PI—protease inhibitor; TDF—Tenofovir; VF—virological failure (defined as viral load ≥1000 copies/mL); 3TC—Lamivudine. [1]Relative risks were estimated using a Poisson regression model with robust standard errors; adjusted models included age, history of virological failure within two years prior to study, base ART regimen at index DRM result, age of caregiver, caregiver viral suppression, adherence, caregiver depression, medicine administration confidence, time to facility, and clinic volume. The observations with missing data were excluded; the extent of missingness is summarized in Table 1.

[2]The relative risk cannot be calculated when the frequency of one of the categories is 0 which leads to quasi-complete separation and prevents proper estimation

[3]Not included in adjusted models due to collinearity with base ART regimen at index DRM result.

Bold text indicates estimates with p-values < 0.05.
